# Endoscopic transventricular resection of a colloid cyst

**DOI:** 10.3171/2023.1.FOCVID22140

**Published:** 2023-04-01

**Authors:** Sebastian Lehmann, Henry W. S. Schroeder

**Affiliations:** Department of Neurosurgery, University Medicine Greifswald, Germany

**Keywords:** colloid cyst, endoscopic resection, navigation, ventriculoscope

## Abstract

This video demonstrates the purely endoscopic gross-total resection of a third ventricle colloid cyst that is partially covered by a large thalamostriate vein. To gain an ideal approach, the initial trajectory pointed to the interventricular septum above the cyst. After the head of the caudate nucleus is passed, it is retracted laterally by the endoscopic sheath for the ideal far lateral approach to the cyst. Using a pneumatic endoscope holder enables the surgeon to perform the procedure bimanually. After complete removal of the cyst, postoperative inspection confirms the intact fornix, veins, and caudate nucleus without signs of pressure-related damage.

The video can be found here: https://stream.cadmore.media/r10.3171/2023.1.FOCVID22140

## Transcript

This case addresses the endoscopic transventricular resection of a colloid cyst of the third ventricle. On admission, the patient suffered from headache and intermittent nausea. In the preoperative MRI, you can see the third ventricle colloid cyst with a dilated right lateral ventricle.

We decided to perform a purely endoscopic navigation-guided transventricular resection.

### 0:45 Patient Prepared for Surgery.

For preoperative preparation, planning of the trajectory was carried out based on a thin-sliced T1-weighted MRI. Our aim was to gain an optimal trajectory, reaching the colloid cyst at the foramen of Monro from as far lateral as possible. To achieve this approach, the initial trajectory is pointed not to the cyst itself, but further medial to the interventricular septum. Once the head of the caudate nucleus is passed, the trajectory is shifted downward to reach the colloid cyst.^1^ We chose to access the cyst via the dilated right ventricle, where the cyst lies directly at the foramen of Monro. In the preoperative MRI, you already see a very prominent thalamostriate vein, rendering this case particularly challenging.

For endoscopic intraventricular procedures, we use the LOTTA system in combination with a pneumatic endoscope holder.^2^

For the approach, the patient is placed in a supine position. The head is slightly anteflected, so the planned burr hole is the highest point in order to minimize postoperative pneumocephalus. The correct entrance point is located with the help of our neuronavigation. For aesthetic reasons, the skin is incised in a straight fashion right behind the hairline.

### 1:55 Surgical Procedure.

A burr hole is placed and the navigation-guided sheath for the endoscope is carefully inserted, following the preplanned trajectory, reaching the lateral ventricle with direct view to the interventricular septum and the head of the caudate nucleus. Once the caudate nucleus head is covered by the endoscope sheath, the sheath is used as a retractor to dislocate the head of the caudate nucleus a little bit laterally to get the ideal approach to the colloid cyst’s attachment at the roof of the third ventricle. The anatomical landmarks for orientation are the fornix, the choroid plexus, as well as the thalamostriate and the septal veins. This case is complicated by the high-caliber thalamostriate vein partially covering the cyst. The main difficulty here is to mobilize the cyst into the lateral ventricle, without injuring the thalamostriate vein or the fornix, both of which would result in severe consequences.

At first, the choroid plexus covering the cyst’s surface is coagulated. After sharp incision, the content of the cyst is removed with a suction tube. Then the cyst is mobilized into the lateral ventricle. Using the pneumatic endoscope holder enables the surgeon to bimanually dissect the colloid cyst, exposing the cyst’s pedicle attached to the tela choroidea.

### 3:10 Removal of Tumor.

Bimanually, the vascularized pedicle is coagulated and sharply dissected from the choroid plexus.^3^ Subsequently, the cyst can be removed.

After complete removal of the cyst, the operative field is inspected. Here, you see the choroid plexus, where the cyst was attached; the intact fornix; and the unharmed caudate nucleus. Usually, we check the contralateral foramen of Monro and the aqueduct for blood clots, which may cause obstructive hydrocephalus. Here, this measure was waived due to the very narrow foramen of Monro to avoid injury to the fornix.

Postoperatively, the patient is doing fine. In the postoperative MRI, the approaching canal placed relatively lateral can be seen without any caudate nucleus pressure-caused injury.

### 3:53 Postoperative Course.

In conclusion, a purely endoscopic procedure via the LOTTA ventriculoscope using a bimanual sharp dissection technique enables a gross-total resection of most colloid cysts.

## Disclosures

Dr. Schroeder reported personal fees from Karl Storz SE & Co. KG, Tuttlingen, Germany, during the conduct of the study.

## Author Contributions

Primary surgeon: Schroeder. Editing and drafting the video and abstract: Lehmann. Critically revising the work: Schroeder. Reviewed submitted version of the work: Schroeder. Approved the final version of the work on behalf of both authors: Lehmann. Supervision: Schroeder.
